# Identification of a novel splice site mutation in the *SERAC1* gene responsible for the MEGDHEL syndrome†

**DOI:** 10.1002/mgg3.815

**Published:** 2019-06-28

**Authors:** Sarah Snanoudj, Patrick Mordel, Quentin Dupas, Cécile Schanen, Alina Arion, Marion Gérard, Marie‐Hélène Read, Djamel Nait Rabah, Didier Goux, Françoise Chapon, Mickael Jokic, Stéphane Allouche

**Affiliations:** ^1^ Departments of Biochemistry University Hospital of Caen Caen France; ^2^ Department of Medical genetics University Hospital of Caen Caen France; ^3^ Department of Signalisation, Électrophysiologie et Imagerie des Lésions d'Ischémie‐Reperfusion Myocardique Normandie Univ, UNICAEN Caen France; ^4^ Department of Medical pediatrics University Hospital of Caen Caen France; ^5^ Department of Medical imaging University Hospital of Caen Caen France; ^6^ CmaBio3, SF 4206 ICORE, Normandie Univ, UNICAEN Caen France; ^7^ Department of Pathology University Hospital of Caen Caen France; ^8^ Department of Medico‐Surgical Pediatric Intensive Care Unit University Hospital of Caen Caen France

**Keywords:** 3‐MGA, cardiolipin metabolism, MEGDHEL, mitochondria, *SERAC1*

## Abstract

**Background:**

MEGDHEL is an autosomal recessive syndrome defined as 3‐MEthylGlutaconic aciduria (3‐MGA) with Deafness, Hepatopathy, Encephalopathy, and Leigh‐like syndrome on magnetic resonance imaging, due to mutations in the *SERAC1* (Serine Active Site Containing 1) gene, which plays a role in the mitochondrial cardiolipin metabolism.

**Methods:**

We report the case of a young patient who presented with a convulsive encephalopathy, 3‐methylglutaconic aciduria, deafness, and bilateral T2 hypersignals of the putamen and the thalami, who passed away at 8 years of age.

**Results:**

Analysis of nuclear genes using an ampliSeq^™^ targeted custom panel disclosed two compound heterozygous variants in the *SERAC1* gene: a nonsense substitution in exon 4, c.202C>T, resulting in a premature stop codon (p.Arg68*), and a novel variant at a canonical splicing site upstream exon 4 (c.129‐1G>C). mRNAs sequencing from the fibroblasts of the patient showed that the splice site variant resulted in exon 3 skipping without frameshift while Western blot experiments showed the absence of SERAC1 expression compared to controls and abnormal filipin staining.

**Conclusion:**

We showed that the loss of the putative transmembrane domain of SERAC1, due to a novel splice site variant, impairs the protein expression and is responsible for the MEGDHEL syndrome.

## 1 INTRODUCTION

Cardiolipins (CL), or diphosphatidyl glycerol, are phospholipid dimer composed of two phosphatidic acids bridged by a glycerol molecule. They are mainly found in the inner mitochondrial membrane (Lu & Claypool, 2015) where they are thought to participate in the cristae formation and ATP production by both electrostatic and hydrophobic interactions with the protein complexes of the respiratory chain (Musatov & Sedlák, 2017). As recently confirmed (Oemer et al., 2018), there is a vast diversity in CL composition across different tissues, with probably functional consequences in terms of energy production. CL remodeling, operated by different lipases followed by acyltransferases, enables modification of acyl chains composition to obtain symmetry between the two phosphatidyl groups and a higher degree of unsaturation (Lu & Claypool, 2015). The importance of such process is highlighted by mutations in the *TAZ* gene, encoding a phospholipid‐lysophospholipid transacylase, which cause Barth syndrome (OMIM ID: #302060, former 3‐methylglutaconic aciduria type II), an X‐linked disorder characterized by a dilated cardiomyopathy, cyclic neutropenia, muscle hypotonia, 3‐methylglutaconic aciduria (3‐MGA‐uria), and mitochondrial alterations. In 2012, Wortmann et al. identified disease‐causing variants in *SERAC1* (Serine Active Site Containing 1, OMIM ID: *614725), encoding a protein containing a serine‐lipase domain with putative phosphatidylglycerol (PG) remodeling activity (Wortmann et al., 2012). Mutations in this gene are responsible for the MEGDHEL syndrome (OMIM ID: #614739), which is an autosomal recessive disorder defined as 3‐MGA‐uria with deafness, hepatopathy, encephalopathy, and Leigh‐like syndrome on magnetic resonance imaging (MRI). Other clinical signs include progressive spasticity and dystonia, failure to thrive, developmental delay or regression, optic nerve atrophy, epilepsy, microcephaly, dysmorphia, and renal tubulopathy, but are not found systematically. Blood tests can also show neonatal hypoglycemia, liver damage with elevation of transaminases associated with hyperbilirubinemia and lactic acidosis. Inconstant impairments of the mitochondrial oxidative phosphorylation (OXPHOS) have also been reported in muscle or liver biopsies, or in fibroblasts. A recent multicentric study summarized the clinical course of the disease in 67 patients with MEGDHEL syndrome and reported the different pathological variants identified in *SERAC1* until now (Maas et al., 2017).

Here we report a novel mutation at a canonical splicing site, associated with an already described missense mutation, of *SERAC1* gene in a young patient. We demonstrate that this original variant is pathogenic and results in exon 3 skipping without frameshift. The loss of the putative transmembrane domain of SERAC1, due to the novel splice site variant, impairs the protein expression, intracellular cholesterol trafficking and is responsible for the MEGDHEL syndrome.

## 2 METHODS

### 2.1 Ethical compliance

The study was conducted in accordance with ethical standards of our institution and with the principles of the Declaration of Helsinki. Written informed consent was obtained from the parents of the patient.

### 2.2 Description of the patient

The proband was the third male child of nonconsanguineous Caucasian healthy parents. The first child was a healthy girl, and the second a boy with language delay. The mother reported two spontaneous miscarriages. The patient was born at term following a pregnancy without abnormalities detected on routine ultrasound, but marked by maternal anorexia. Birth weight and height were 2,890 g (−1 *SD*) and 46.5 cm (−1.5 *SD*), respectively, and head circumference was 35 cm (+0.5 *SD*), with Apgar scores of 10 at 1 and 5 min. The first reported clinical manifestations were a failure to thrive from 5 months old, with developmental delay and hypotonia. At 8 months old, he was hospitalized for the follow‐up of a convulsive encephalopathy. He had an episode of hypoglycemia (0.36 g/L, 2 mmol/L) under fasting conditions, with lactic acidosis (6.7 mmol/L). After feeding every 4 hr, there was a rapid recovery of the weight curve and changes in behavior, with a more active state. A CT‐scan (computed tomography) of the brain showed hypodensities of the basal ganglia.

At 1 year of age, a deltoid muscle biopsy was performed due to suspicion of a mitochondrial disease, but showed nonspecific changes (diffuse atrophy of type II fibers without ragged red fibers or decrease of cytochrome c oxidase staining, Figure S1) without structural alteration of mitochondria in electronic microscopy (data not shown). With the parents’ consent, blood was drawn for DNA extraction, and a skin biopsy was performed for further investigations.

**Figure 1:**
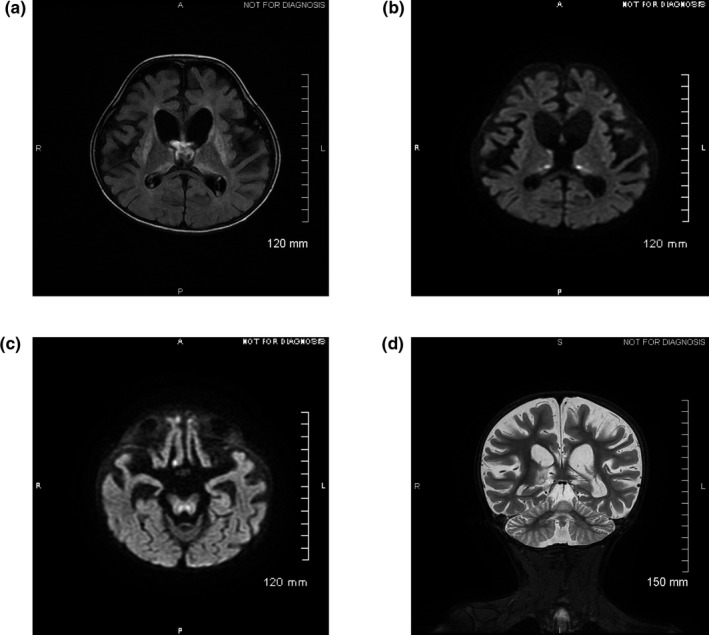
Cerebral magnetic resonance imaging performed at 4 years of age. Axial (a–c) and coronal planes (d). (a) FLAIR sequence shows hypersignals of the basal ganglia and thalami. (b) and (c) reveal hypersignals in thalami and brainstem on diffusion‐weighted imaging. (d) T2‐weighted imaging indicates global cortico‐subcortical atrophy. FLAIR, fluid attenuated recovery

At 4 years old, he could hold his head but not sit, had no voluntary grip and did not speak. Since he did not react to loud sounds, an automated otoacoustic emission test was performed and confirmed hearing loss, with no reproducible response from the peripheral level. Ophthalmological assessment showed bilateral optic atrophy. He presented with failure to thrive and microcephaly, with a height of 98 cm (−2 *SD*), a weight of 11.380 kg (−3 *SD*), and a head circumference of 48 cm (−1.5 *SD*). Clinical examination revealed major axial hypotonia and mild dystonia. Organic acid analysis in the urine demonstrated increased excretion of both 3‐methylglutaconic acid and 3‐methylglutaric acid; peaks were not quantified but ranged from 100 to 200 mmol/mol of creatinine (normal < 20 mmol/mol of creatinine). Although this was a significant excretion, it was not enough to suspect a primary 3‐MGA‐uria (usually > 400 mmol/mol of creatinine, Wortmann, Kluijtmans, Sequeira, Wevers, & Morava, 2014). Brain MRI (Figure 1a–d) showed a major and diffuse cortical and subcortical atrophy affecting both the infratentorial and supratentorial regions, bilateral abnormalities of the putamen and thalami with hyperintense signals on the T2‐, fluid attenuated recovery‐ and diffusion‐weighted images, and hypointense signals on the T1‐weighted images consistent retrospectively with the Leigh‐like syndrome typically found in MEGDHEL patients (Wortmann et al., 2015). Brain MR spectroscopy showed increased lactate, with high levels in the putamen, and reduced N‐acetyl‐aspartate, a marker of neuronal loss. At 7 years of age, because of a low weight (−2.5 *SD*) and feeding difficulties, he underwent antireflux surgery combined with gastrostomy placement, and salivary gland botulinum toxin injections for excessive salivation and drooling. It is worthy to note that a minor hemophilia A was discovered at that time, which required replacement therapy. At 8 years of age, he underwent general anesthesia for dental extraction and experienced severe acute respiratory distress syndrome postoperatively. Brain MRI showed major systemic atrophy, hyperintense T2 signals of the putamen and hyperintense T2 signals of the brain stem, signing acute brain injury. He was extubated and passed away with no diagnosis.

### 2.3 Next generation sequencing

Genomic DNA extracted from peripheral blood was analyzed by next generation sequencing (NGS) on a custom‐designed panel of nuclear genes with mitochondrial function. The Ion AmpliSeq^™^ designer software was used to design primers for multiplex PCR amplification of the selected 38 nuclear genes (441 exons with 50 bp exon padding). Libraries were constructed using the Ion AmpliSeq Library Kit v2.0 (Life Technologies, Carlsbad, CA, USA) according to the manufacturer's instructions. Quantification of enriched target DNA was performed on the Qubit^™^ 2.0 Fluorometer using the Qubit^™^ dsDNA HS Assay Kit (Life Technologies).

Amplified libraries were submitted to emulsion PCR on the Ion OneTouch^™^ 2 system using the Ion PGM^™^ Hi‐Q^™^ View OT2 Kit (Life Technologies) according to the manufacturer's instructions. Ion sphere particles (ISP) were enriched using the E/S module. Resultant live ISPs were loaded and sequenced on an Ion 316 chip (Life Technologies) using an Ion Torrent PGM^™^ Sequencer.

Ion Reporter^™^ and NextGENe^®^ v.2.4.1.2 (SoftGenetics, State College, PA, USA) softwares were further used to perform primary to tertiary analyses using the hg19 reference genome, including optimized signal processing, base calling, sequence alignment, and variant analysis. Pathogenic and likely pathogenic variants were further verified by conventional dideoxy sequencing (Sanger sequencing) using the BigDye^®^ Terminator v.1.1 Cycle Sequencing Kit (Life Technologies). After purification, sequencing products were applied onto an ABI 3500 Genetic Analyzer (Life Technologies).

### 2.4 RT‐PCR analyses

Total RNAs were extracted from either control or patient skin fibroblasts using the TRIzol reagent (Promega, Madison, Washington, USA). Control fibroblasts were obtained from patients with a suspicion of neuromuscular disease but whose genetics investigations came back negative, including *SERAC1* sequencing. cDNAs were synthesized from 3 μg of RNA using the reverse transcriptase system (Promega). Primers for glyceraldehyde‐3‐phosphate dehydrogenase (control) and *SERAC1* (NM_0.032861.3) were designed using Primer3 Input (Koressaar & Remm, 2007; Untergasser et al., 2012). PCRs were performed using 2 μL of cDNAs in the PCR buffer supplied with the Taq polymerase supplemented with 1.5 mmol/L MgCl_2_, 0.2 mmol/L of dNTP, 2.5 U of Taq polymerase (Bioline, London, UK), and 0.5 mmol/L of each sense and antisense primers.

### 2.5 Enzymatic activity determinations and ATP measurement

Respiratory chain enzyme activities in skeletal muscle homogenate and in isolated mitochondria from fibroblasts were determined spectrophotometrically as previously described (Medja et al., 2009).

### 2.6 Western blot

Whole cell lysates from fibroblasts of the patient and controls were obtained after a 10 min incubation at 4°C in 20 mmol/L Tris‐HCl pH 7.4, 150 mmol/L NaCl, 1 mmol/L EDTA, and 1% (v:v) triton X‐100. Lysates were cleared by centrifugation at 20,000 *g* during 20 min at 4°C, then proteins were subjected to SDS‐PAGE and electroblotted onto a nitrocellulose sheet. Immunoreactive bands were detected using anti‐SERAC1 (Sigma‐Aldrich, Saint‐Louis, MO, USA) and anti‐actin (Abcam, Cambridge, UK) antibodies followed by horseradish peroxidase‐conjugated secondary antibodies (Cell signaling technology, Leiden, The Netherlands), and visualized by enhanced chemiluminescence (Western Lightning Chemiluminescence Reagent Plus, PerkinElmer, Waltham, MA, USA).

### 2.7 Filipin staining

Intracellular unesterified cholesterol was visualized using the cholesterol assay kit (Abcam) according to the manufacturer's instructions. Thirty thousand fibroblasts were grown on glass coverslips for 2 days in culture medium. Then, fibroblasts were either exposed to the cholesterol trafficking inhibitor U‐18666A at 2.5 μmol/L, or to DMSO for 48 hr. Cells were washed, fixed, and stained with Filipin III for 60 min at room temperature in the dark. After additional washes, coverslips were mounted and images were obtained using a Zeiss microscope and Axiovision software with a x60 lens (Zeiss, Jena, Germany).

## 3 RESULTS

**Figure 2:**
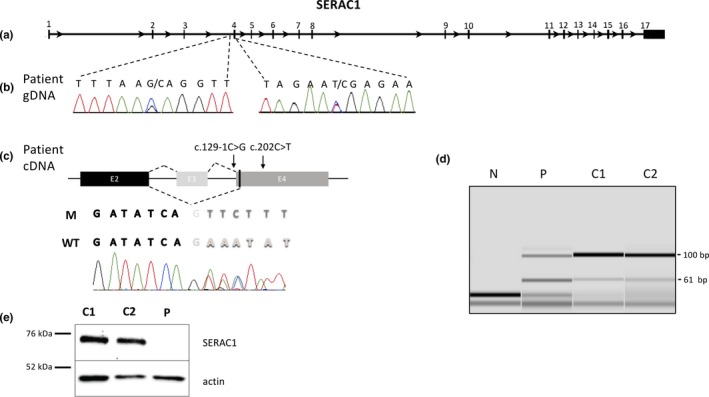
Effect of the two variants in the *SERAC1* gene at the mRNA and protein levels. (a) Representation of the *SERAC1* gene (Gene ID: 84947, NCBI). Exons are numbered from 1 to 17 and are represented as green boxes. (b) Identification of the two variants in the *SERAC1* gene from the gDNA of the patient. (c) Sequencing of the cDNA from the patient's fibroblasts. Exons 2, 3, and 4 are represented as black, light gray, and dark gray boxes, respectively. Splicing from controls generated the WT sequence while we observed the exon 3 skipping in the patient with the mutated (M) sequence; black letters correspond to the end of exon 2, light gray letters to the beginning of the exon 3 and dark gray letters to the beginning of the exon 4 and bold letters correspond to heterozygosity. (d) Capillary electrophoresis of PCR products from fibroblast mRNAs. Primers, located at the end of exon 2 and at the beginning of exon 4, enable amplification of a 100 bp product corresponding to the wild type mRNA, and a 61 bp product corresponding to exon 3 skipping. In two controls (C1 and C2), the 61bp‐band can be seen at a 1:10 ratio compared to the WT, indicating that there is a minor splicing site resulting in exon 3 skipping, but with no corresponding protein described. In the patient (P) both products are present at a 1:1 ratio, and an additional band can be found above the 100bp‐band, signing the presence of heteroduplexes. (e) SERAC1 protein expression was studied by Western blot from the patient's fibroblasts (P) and two controls (C1 and C2). Wild type SERAC1 protein is detected at about 74 kDa, and actin was used as a loading control. WT, wild type

Our custom gene panel allowed us to analyze both coding sequences as well as the 5′ and 3′ flanking regions of 38 nuclear genes with a mean depth of 586X. Using two different pipelines, we identified seven variants with a minor allele frequency <1%. Five were located in intronic regions and were reported as rare polymorphisms. The other two variants were found in the *SERAC1* gene (NM_0.032861.4): one nonsense substitution in exon 4, NG_032889.1(*SERAC1*_v001): c.202C>T, resulting in a premature stop codon (NP_116250.3; p.(Arg68*)), and the other substitution at a canonical splice site upstream the exon 4, (NG_032889.1(*SERAC1*_v001):c.129‐1G>C, Figure 2b). A close examination of reads obtained from NGS analysis indicated that the two variants were in *trans* positions (Figure S2). The nonsense c.202C>T variant was previously reported as a disease‐causing homozygous variant in a patient presenting with a MEGDHEL syndrome (Tort et al., 2013). The other substitution (i.e., c.129‐1G>C) was not found in multiple databases (Genome Aggregation Database, Exome Variant Server, dbSNP), and, to the best of our knowledge, has not been previously described in the literature. A splice effect of the variant c.129‐1G>C was predicted by two independent in silico splicing predictions tools, MaxEntScan (Yeo & Burge, 2004) and Human Splicing Finder 3.0 (Desmet et al., 2009). Analysis of mRNA from the patient's fibroblasts by RT‐PCR and subsequent sequencing indicated that the splicing site variant results in the skipping of exon 3 (PCR product of 61 bp vs. 100 bp for the wild type [WT]) and a new acceptor site at the beginning of exon 4 (c.131), resulting in a predicted protein with 14 amino acids deleted (codons 31 to 44, RNIIKFTGSLILGG), and the insertion of a serine, without a frameshift (Figure 2c,d).

To evaluate the consequences of the two *trans* variants detected in our patient, we further studied the SERAC1 protein by Western blot. We observed a specific band at the expected size of about 74 kDa in fibroblasts from controls but not in the patient, while actin was detected in all fibroblast lysates (Figure 2e).

Examination of the mitochondrial respiratory chain was conducted by spectrophotometric analysis in both skeletal muscle and skin fibroblasts but did not reveal any deficiency (data not shown). However, we were able to show a significant reduction by 60% in the ATP production compared to controls. We also ruled out any mutation in the mitochondrial DNA (mtDNA) by NGS and deletions/rearrangements by long PCR and Southern‐blot (data not shown).

**Figure 3:**
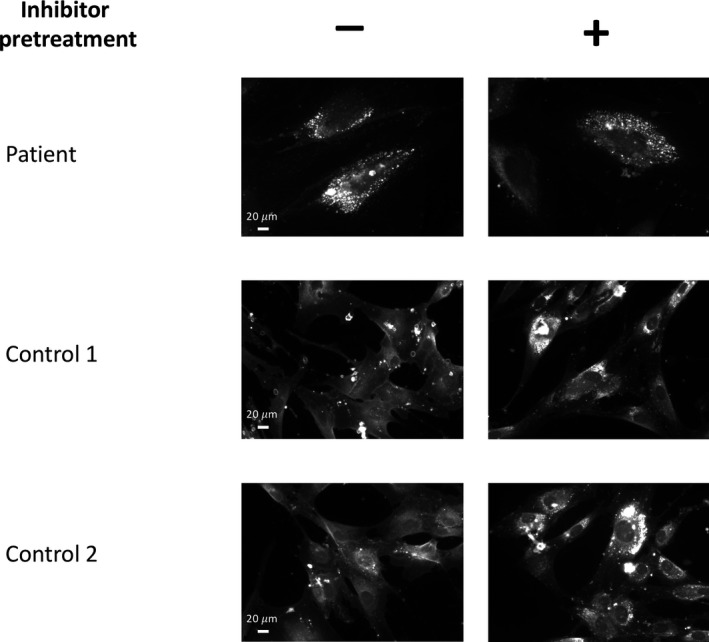
Defective cholesterol trafficking in fibroblasts carrying *SERAC1* variations. The fibroblasts from the patient carrying the *SERAC1* variations and controls were grown on coverslips and exposed or not to a cholesterol trafficking inhibitor (positive control). Cells were stained with Filipin III and cholesterol was visualized by fluorescence microscopy at a 60X lens

Since SERAC1 protein was demonstrated to be involved in intracellular cholesterol trafficking (Wortmann et al., 2012), we assessed the unesterified cholesterol distribution in fibroblasts using filipin staining. In the patient's fibroblasts, we observed a fluorescence staining as strong as detected in control fibroblasts with a cholesterol‐trafficking inhibitor, U18666A (Figure 3).

## 4 DISCUSSION

Here, we describe the case of a patient with a convulsive encephalopathy who passed away at the age of 8 years old with no genetic diagnosis. After ruling out mutations and deletions/rearrangement of the mtDNA, molecular analysis of nuclear genes involved in mitochondrial diseases by NGS revealed that the patient was a compound heterozygote for two variations in the *SERAC1* gene: a nonsense substitution previously described as disease‐causing (Tort et al., 2013) and a novel splice site variant resulting in exon 3 skipping. The *SERAC1* gene is composed of 17 exons (the first exon is noncoding, Figure 2a) and the main transcript (NM_032861.3) encodes a protein of 654 amino acids (NP_116250.3) with a single transmembrane domain (32–54) and a serine‐lipase domain (396–540). This protein is located at the interface of mitochondria and endoplasmic reticulum and could play a critical role in PG remodeling (Wortmann et al., 2012). Most of the disease‐associated variants reported in the N‐terminal domain of SERAC1 result in truncated protein without the lipase activity domain (Maas et al., 2017). In the present study, we describe a novel variant that results in exon 3 skipping with a 14 amino acid deletion (31–44) in the transmembrane domain but without frameshift. Surprisingly, we were unable to detect SERAC1 expression in fibroblast by Western blot suggesting that both the nonsense and the splice site variants would trigger mRNA degradation. While the role of the transmembrane domain of SERAC1 is not clearly defined, we can hypothesize that this region could enable anchoring of this enzyme in the membranes of both mitochondria and endoplasmic reticulum. Using the PredictProtein resource (Yachdav et al., 2014), we observe that the predicted transmembrane region (33–58) in the WT protein would be disrupted by the 14 amino acids deletion.

*SERAC1* mutations were demonstrated to change acyl‐chain composition of PG, a precursor of CL; this results in normal levels of CL but with altered acyl chain composition. The consequences on mitochondrial functions are still unclear, since alterations of the respiratory chain were reported in only about 40% of patients with *SERAC1* mutations, in muscle biopsies or in fibroblasts (Maas et al., 2017). While we did not demonstrate significant decrease in OXPHOS in these tissues (complexes I‐IV), we showed a reduction in ATP production. This could be explained by different experimental conditions for enzyme and ATP measurements. It could also be explained by a complex defect of the respiratory chain and in particular of the ATP synthase (not measured by spectrophotometric analysis). SERAC1 defects also reduce the levels of bis(monoacylglycerol)phosphate which regulates cholesterol trafficking (Wortmann et al., 2012). As previously reported by others (Rodríguez‐García et al., 2016; Roeben et al., 2018; Sarig et al., 2013; Tort et al., 2013; Wortmann et al., 2012), filipin staining was abnormal in the patient's fibroblasts.

Retrospectively, the patient fulfilled most of the criteria for diagnosis of MEGDHEL syndrome (Table 1): a 3‐MGA‐uria, deafness, encephalopathy, and a Leigh‐like syndrome on MRI. Other signs present in our patient are also described in the literature and include neurological regression and psychomotor retardation, spasticity, dystonia, failure to thrive, feeding difficulties, epilepsy, drooling, microcephaly, optic atrophy, and hypoglycemia. Because of the absence of OXPHOS deficiency and since the genetic investigations available at that time yielded no result, the diagnosis was delayed, despite 3‐MGA urinary excretion and suspicion of a mitochondrial disorder. Moreover, as MEGDHEL is such a rare syndrome, the association of symptoms was not well recognized. In 2013, Sarig et al. described infantile hepatopathy as a cardinal feature of the MEGDEL syndrome, which was renamed MEGDHEL, but there was no sign of hepatopathy in our patient (Sarig et al., 2013). This is not surprising as liver dysfunctions are generally transient and resolve spontaneously.

**Table 1:** Comparison between the previously reported patients with MEGDHEL syndrome and mutations in *SERAC1,* and the patient described here

	Patient	Literature	
		*N*	%
Consanguinity	−	44/66	67
Psychomotor regression	+	55/61	90
Age at regression	6 mo	birth to 4 yo (median 12 mo)	
Spasticity	+	59/72	82
Dystonia	+	60/73	82
Deafness	+	54/68	79
Failure to thrive	+	21/21	100
Epilepsy	+	29/74	39
Microcephaly	+	10/10	100
Neonatal liver dysfunction	NA	38/70	54
Optic atrophy	+	14/57	25
Leigh‐like syndrome on brain MRI	+	65/66	98
3‐MGA‐uria	+	72/73	99
Range (mmol/mol of creatinine)	>20	20–420	
Lactic acidosis	+	62/72	86
OXPHOS dysfunction M	−	11/20	55
OXPHOS dysfunction F	−	3/11	27
Positive filipin staining in F	+	10/14	71

Note

F, fibroblast; M, muscle; mo, months old; MRI, magnetic resonance imaging; *N*, number; NA, not available; OXPHOS, oxidative phosphorylation; Yo, years old; +, present; −, absent. Literature data obtained from Maas et al.

Sixty‐seven patients with a MEGDHEL syndrome have recently been reviewed in the literature with a diagnosis confirmed by the presence of pathogenic variants in the *SERAC1* gene. Forty‐one different variants have been described: some are found in multiple families, but most of them are private ones (Maas et al., 2017). Clinical presentations of the MEGDHEL syndrome are strikingly similar, with an invariably severe phenotype, with one notable exception recently described (Roeben et al., 2018). These authors reported six affected subjects of a consanguineous family with a novel splice variant downstream of exon 2, who presented with a juvenile‐onset complicated hereditary spastic paraplegia (cHSP). The phenotype was much milder compared to MEGDHEL syndrome, but patients also present with typical findings such as the “putamen eye” on brain MRI (Wortmann et al., 2015), and 3‐MGA‐uria. This milder phenotype correlates with minor changes of PG (34:1)/PG (36:1) ratio and no abnormal filipin staining, further expanding the phenotypic spectrum of SERAC1 deficiency, with two extreme clusters: a severe, infantile onset “MEGDHEL” phenotypic cluster and a slowly progressive oligoasymptomatic “cHSP” cluster beginning during adolescence, but with no intermediate forms.

In conclusion, we report here a novel splice site variant in the *SERAC1* gene that can be classified as pathogenic according to the ACMG standards and guidelines (Richards et al., 2015), with one very strong (PVS1), one strong (PS3), two moderate (PM2, PM3), and one supporting (PP4) evidences of pathogenicity. Our data also highlight the importance of the clinical course, the cerebral imaging and the biological analyses to select patients for the molecular testing of *SERAC1*, as well as the role of gene panels in mitochondrial disorders.

## CONFLICT OF INTEREST

The authors (SS, PM, QD, CS, AA, MG, MHR, DNR, DG, FC, MJ, and SA) declare that they have no conflict of interest.
